# Integration of sexually transmitted infection and HIV pre-exposure prophylaxis services in sub-Saharan Africa: a scoping review

**DOI:** 10.3389/frph.2023.944372

**Published:** 2023-06-29

**Authors:** Priyanka Anand, Linxuan Wu, Kenneth Mugwanya

**Affiliations:** ^1^ Department of Medicine, University of Washington School of Medicine, Seattle, WA United States; ^2^ Department of Global Health, University of Washington, Seattle, WA, United States; ^3^ Department of Epidemiology, University of Washington, Seattle, WA, United States

**Keywords:** STIs—sexually transmitted infections, PrEP (pre-exposure prophylaxis), sub sahara Africa, service integration, HIV prevention, STI prevention

## Abstract

**Background:**

Persons living in sub-Saharan Africa (SSA) face disproportionate risk from overlapping epidemics of HIV and bacterial sexually transmitted infections (STIs). Pre-exposure prophylaxis (PrEP) for prevention is gradually being scaled up globally including in several settings in SSA, which represents a key opportunity to integrate STI services with HIV pre-exposure prophylaxis (PrEP). However, there is limited literature on how to successfully integrate these services, particularly in the SSA context. Prior studies and reviews on STI and PrEP services have largely focused on high income countries.

**Methods:**

We conducted a scoping review of prior studies of integration of STI and PrEP services in SSA. We searched PubMed, EMBASE, Cochrane, and CINAHL, in addition to grey literature to identify studies that were published between January 2012 and December 2022, and which provided STI and PrEP services in SSA, with or without outcomes reported. Citations and abstracts were reviewed by two reviewers for inclusion. Full texts were then retrieved and reviewed in full by two reviewers.

**Results:**

Our search strategy yielded 1951 records, of which 250 were retrieved in full. Our final review included 61 reports of 45 studies. Most studies were conducted in Southern (49.2%) and Eastern (24.6%) Africa. Service settings included public health clinics (26.2%), study clinics (23.0%), sexual and reproductive care settings (23.0%), maternal and child health settings (8.2%), community based services (11.5%), and mobile clinics (3.3%). A minority (11.4%) of the studies described only syndromic STI management while most (88.6%) included some form of etiological laboratory STI diagnosis. STI testing frequency ranged from baseline testing only to monthly screening. Types of STI tested for was also variable. Few studies reported outcomes related to implementation of STI services. There were high rates of curable STIs detected by laboratory testing (baseline genitourinary STI rates ranged from 5.6–30.8% for CT, 0.0–11.2% for GC, and 0.4–8.0% for TV).

**Discussion:**

Existing studies have implemented a varied range of STI services along with PrEP. This range reflects the lack of specific guidance regarding STI services within PrEP programs. However, there was limited evidence regarding implementation strategies for integration of STI and PrEP services in real world settings.

## Introduction

Persons living in sub-Saharan Africa face disproportionate risk from overlapping epidemics of HIV and bacterial STIs. Specifically, young African women face a disproportionate risk of HIV acquisition, accounting for more than half of new infections on that continent, with incidence rates that are often more than double that of their male age-mates (1). At the same time, African women also face a disproportionate burden of sexually transmitted infections (STIs). Globally, the World Health Organization (WHO) estimates that 358 million new cases of four curable sexually transmitted infections with the greatest burden in low- and middle-income countries (2). The overlapping epidemics of HIV and bacterial STIs in Africa have been recognized since the earliest days of the HIV epidemic.

The WHO has recommended greater bi-directional integration of STI and PrEP programs, by both incorporating STI services into PrEP programs and targeting STI clients as potential PrEP clients. Furthermore, they recommend moving beyond a syndromic approach to diagnostic STI testing and treatment, given concerns related to missed diagnoses and overtreatment (2). National PrEP guidelines in a number of countries in SSA that have rolled out PrEP recommend STI screening at baseline and in follow up; this is largely done via syndromic management due to limited resources (3–5). A number of barriers exist to implementation of STI testing and service delivery in SSA, including financial, logistical and time constraints. Despite the need for innovative approaches to providing combined STI and PrEP services, limited literature exists around models of integration of STI and PrEP programs (6, 7).

We sought to investigate the evidence around integration of PrEP services with STI services in the SSA context via a scoping review of the literature. Specifically, we aimed to better understand in what contexts STI and PrEP integration had been studied, what types of STI services had been integrated with PrEP, and what evidence existed around barriers and best practices for integration of these services.

## Methods

We conducted scoping review to evaluate the evidence base for integration of PrEP and STI services in SSA. We used a systematic approach, following Preferred Reporting Items for Systematic Reviews and Meta-Analyses extension for Scoping Reviews (PRISMA-ScR) guidelines (8). A study protocol was drafted and filed prior to the search, and is available online via figshare (9).

A full electronic search strategy covered all studies published between January 2012 and December 2022. Studies prior to 2012 were not included due to the lack of PrEP implementation in SSA during that period. Key words were generated to describe the scoping review concepts using index articles as well as the authors’ background knowledge. Initial search strategies were developed using PubMed advanced search builder, which was then followed by an exploratory search. The final search strategy included four core concepts: (HIV) AND (Pre-Exposure Prophylaxis) AND (Sexually Transmitted Infections) AND (Sub-Saharan Africa). This search strategy was subsequently adopted to search other databases that included EMBASE, Cochrane, and CINAHL. We also searched conference proceedings and abstracts from major international HIV and STI conferences (i.e., IAS, CROI, ISTDR, INTEREST, Adherence) and the grey literature for reports focused on PrEP and STI services delivery in SSA from World Health Organization (WHO), non-governmental organizations (NGO), and intergovernmental organizations (IGO) that were available on the following databases: Union of International Associations IGO Search, IGO/NGO custom search engines, WHO Institutional Repository for Information Sharing (IRIS), and WHO Library Database. A full description of the search strategy is provided in Supplementary Appendix I.

Following the search, all identified citations were collated and uploaded into Rayyan, a collaborative software for systematic reviews, and duplicates were removed (10). English-language records with a focus on PrEP and STI service delivery were included. In line with the review question, we included studies which described provision of PrEP and STI services as well as the outcomes of these services. PrEP services were defined as provision of PrEP as well as clinical follow-up. Studies that only described promotion, education, counseling, HIV risk assessment or referral related to oral PrEP without any provision of PrEP were not included. We limited our review to oral daily PrEP as it is the current available modality of PrEP that has been approved to use in SSA. STI services were considered to include promotion, education, consulting, diagnosis (e.g., syndromic diagnosis, laboratory diagnostics, and point of care testing), treatment, or partner services. PrEP and STI outcomes included any quantitative and qualitative outcomes reported regarding these services. Because the overall goal of this scoping review was to describe existing models of STI and PrEP service integration, we also included protocols of ongoing studies that described integration of these services, even if no outcomes were available.

Titles and abstracts were independently screened by two reviewers (PA, LW) for inclusion in the review and potentially relevant articles were then retrieved in full and assessed for inclusion. If only the abstract was available, effort was made to search for a full text article related to the abstract; if none was available, the abstract was included in our review. Full text records were subsequently screened by two independent reviewers for inclusion. Information on study design, context, target population, PrEP and STI services, relevant outcomes, and conclusions were extracted by one reviewer and reviewed by a second reviewer using a data extraction sheet. Disagreements at each step were resolved through discussion between the two reviewers.

## Results

**Figure 1:**
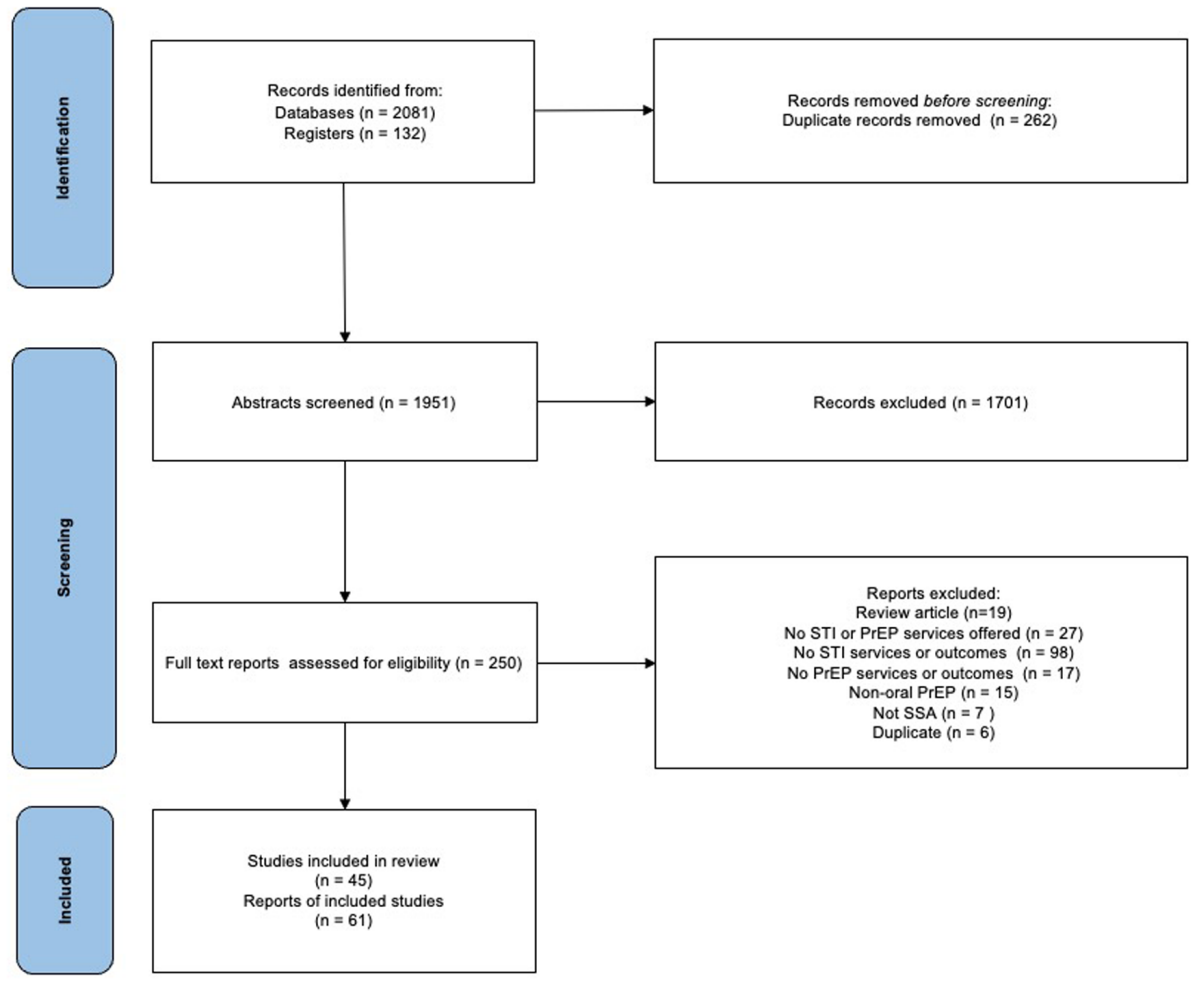
PRISMA flow diagram of study inclusion.

**Figure 2:**
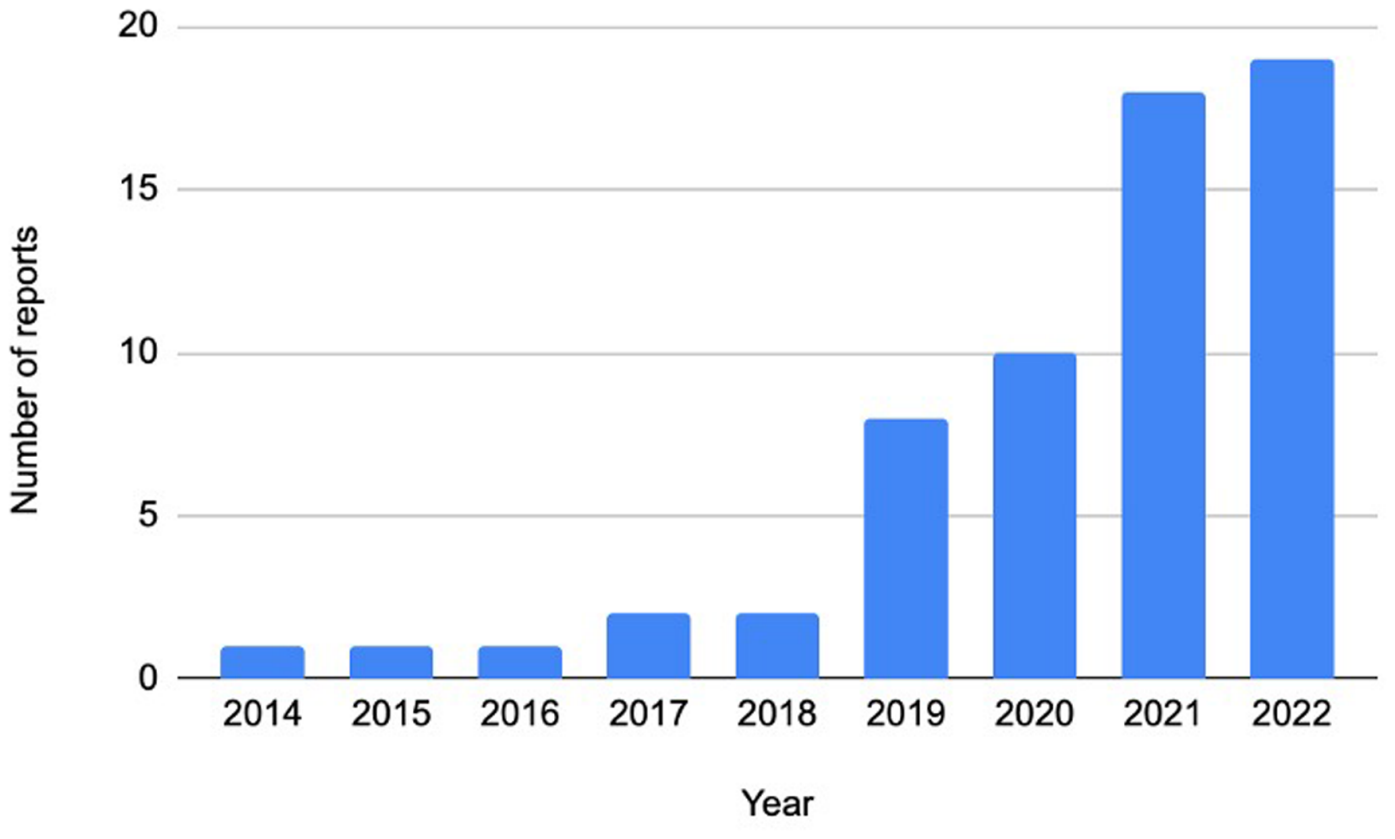
Distribution of year of publication.

After removal of duplicates, the search strategy yielded a total of 1951 records for abstract review, of which 250 were retrieved in full. Of these, 61 reports of 45 studies were selected for inclusion. Of these, 42 (68.9%) were full text reports, 10 (16.4%) were abstracts and 9 (14.8%) were protocols. Details of the records reviewed, including reasons for exclusion at the full text stage, are provided in Figure 1. The majority of papers were published in 2019 or later (88.5%), reflecting the relatively new rollout of PrEP in SSA (Figure 2).

Table 1 shows the characteristics of included reports and type of services and direction of integration. Most papers described cohort studies (34/61, 55.7%), including real-world cohorts and single-arm demonstration studies; randomized control trials (RCTs) comprised a smaller proportion of the included reports (12/61, 19.7%) Consistent with the actual implementation of PrEP in SSA, most studies were conducted in Southern (49.2%) and Eastern (24.6%) Africa, with Western Africa (19.7%) representing a smaller portion of the included reports.

**Table 1:** Summary of included studies.

Study features	*N* (%)
Study Design	
RCT (including cluster RCT)	12 (19.7%)
Cohort	34 (55.7%)
Cross-sectional	4 (6.6%)
Case report	1 (1.6%)
Qualitative	1 (1.6%)
Protocol only	9 (14.8%)
Year of publication	
2022	18 (29.5%)
2021	18 (29.5%)
2020	10 (16.3%)
2019	8 (13.1%)
2018	2 (3.3%)
2017	2 (3.3%)
2016	1 (1.6%)
2015	1 (1.6%)
2014	1 (1.6%)
Region	
Southern	30 (49.2%)
Eastern	15 (24.6%)
Western	12 (19.7%)
Multiple	4 (6.6%)
Population studied	
AGYW	20 (32.8%)
Women	8 (13.1%)
PBFW	3 (4.9%)
Couples	6 (9.8%)
FSW	7 (11.5%)
MSM	7 (11.5%)
Trangender women	1 (1.6%)
Multiple	4 (6.6%)
Youth	2 (3.3%)
Other/General	3 (4.9%)
Context	
Public health/primary care	16 (26.2%)
Sexual and reproductive health	14 (23.0%)
Study clinic	14 (23.0%)
Community based	7 (11.5%)
Maternal and child health	5 (8.2%)
Mobile clinic/venue-based	2 (3.3%)
Multiple	3 (4.9%)
Integration direction	
Both services integrated into a third context	34 (55.7%)
STI services within PrEP program	24 (39.3%)
PrEP services within STI program	3 (4.9%)
STI services described (*n* = 44)^a^	
Laboratory testing at baseline	33 (75.0%)
Laboratory testing at follow up	20 (45.5%)
Syphilis testing only	2 (4.6%)
Syndromic management	13 (29.6%)
Referral for treatment	3 (6.8%)
STI treatment	30 (68.2%)
Partner treatment	2 (4.6%)
STIs and related conditions tested (*n* = 39)^b^	
Chlamydia	30 (76.9%)
Gonorrhea	31 (79.5%)
Trichomonas	17 (43.6%)
Syphilis	21 (53.9%)
Bacterial vaginosis	4 (10.3%)
Candida	1 (2.6%)
Herpes simplex	3 (7.7%)
Mycoplasma genitalum	2 (5.1%)
Hepatitis B	12 (30.8%)
Testing modality (*n* = 39)^c^	
Syphilis serology	13 (29.6%)
Syphilis rapid test	8 (18.2%)
GC NAAT	19 (43.2%)
Gonorrhea gram stain	1 (2.3%)
TV NAAT	6 (13.6%)
TV rapid test	4 (9.1%)
Wet mount miscroscopy	2 (4.6%)
Mycoplasma genitalum multiplex assay	1 (2.3%)
HBV serology	7 (15.9%)
HbsAg rapid test	5 (11.4%)
Not specified	11 (25.0%)
Routine testing frequency (*n* = 39)^c^	
Baseline only	18 (40.9%)
Annual	1 (2.3%)
6 months	12 (27.3%)
3 months	6 (13.6%)
Monthly	2 (4.6%)
Other	2 (4.6%)

GC, N. gonorrheae and C. trachomatis; TV, T. vaginalis; HBV, hepatitis B; HbsAg, hepatitis B surface antigen; NAAT, nucleic acid amplification test; AGYW, adolescent girls and young women; FSW, female sex workers; MSM, men who have sex with men; PBFW, pregnant and breastfeeding women.

^a^ Out of 44 unique study reports (excluding duplicates and studies not reporting specific STI results).

^b^ Out of 39 unique studies conducting laboratory/etiologic testing for STIs (5 studies reporting syndromic screening only were excluded).

^c^ For studies with multiple screening intervals or different screening intervals for different STIs, the shortest interval of screening was used. For example, routine testing at 6, 12 and 24 months was counted as a 6-month interval of screening.

Most papers focused on a particular population at risk for HIV such as adolescent girls and young women (AGYW) (32.8%), female sex workers (FSWs) (11.5%), and men who have sex with men (MSM) (11.5%). Other common populations of focus included women (including peri-conception) (13.1%), pregnant and breastfeeding women (4.9%) and serodiscordant couples (9.8%). Services were implemented in a variety of contexts, including public health clinics (26.2%), study clinics (23.0%), sexual and reproductive care settings (23.0%), maternal and child health settings (8.2%), community based services (11.5%), and mobile clinics (3.3%).

In most cases, both PrEP and STI services were implemented within a third service setting (55.7%). Less frequently, STI services were implemented within a program with a primary focus on PrEP provision, including PrEP RCTs (39.3%). Few studies reported on PrEP services integrated into a primarily STI-focused program (4.9%).

Table 2 provides a summary of data extraction for studies reviewed, including study setting, population, services provides, assays used, and major outcomes. The type of STI services offered varied and often related to the type of study (RCT, demonstration project or real-world implementation). Most studies and study protocols described STI laboratory testing at baseline (75.0%), with fewer describing any follow up laboratory STI testing (45.5%). A quarter described syndromic management, either with or without any etiological testing (29.6%). Type of STIs included in laboratory testing varied. Of 39 studies and protocols that included etiological STI testing, 30 (76.9%) tested for Chlamydia trachomatis (CT), 31 (79.5%) for Neisseria gonorrhoeae (NG), 21 (53.9%) for syphilis, 17 (43.6%) for trichomonas vaginalis (TV), 4 (10.3%) for bacterial vaginosis (BV), 1 (2.6%) for Candida, 2 (5.1%) for Mycoplasma genitalium, and 3 (7.7%) for HSV.

**Table 2:** Data extraction and summary of reviewed studies.

Study/Author	Setting/region	Study population	Context of service point	Integration direction	STI services described	Type of STIs	Assay type used (e.g., ELIZA, NAAT, POC)	Frequency of testing/specimen type	STI outcomes
Benin demonstration project, Mboup et al 2018	Benin	Adult FSWs at risk for HIV	Public health/primary care	Both services integrated into a third context	syphilis, yeasts, TV, TC, NG, BV testing	syphilis, yeasts, TV, BV, NG, CT	SD Bioline rapid test and the RPR test for syphilis; Vaginal swabs for direct microscopy of yeasts, Trichomonas vaginalis, and bacterial vaginosis; NAAT for genital gonococcal and chlamydia.	All STIs at baseline; genital GC and CT tested on longitudianl cervical swabs no explicit testing frequency	Baseline prevalence in PrEP group, NG 11.2%, CT 6%, BV 59.4%,, candidiasis 4.0%, and TV 0.4%. Chlamydia incidence was 4.8/100 person-years
Benin demonstration project, Giguère et al 2019	Benin	Adult FSWs at risk for HIV	Public health/primary care	Both services integrated into a third context	TV, TC, NG; free STI treatment for positives	TV, NG, CT	microscopy for TV; NAAT for NG, CT	Vaginal swab at baseline and every 6 months.	Of 255 participants, 120 (47.1%) completed followup. Prevalence of STI decreased from 15.8% (95% CI: 11.8% to 21.0%) at baseline to 2.1% (95% Cl: 0.4% to 10.2%) at 24
3P, Celum et al 2020	South Africa	HIV-negative non preganant women ages 16 to 25 in a periurban township outside of Capetown	Study clinic	STI services within PrEP program	Test for TV NG, CT	TV, NG, CT	rapid test for TV; NAAT for NG, CT	STI test at baseline with followup testing frequency unspecified	Baseline prevalence of any curable STIs was 32% overall, CT 25%, NG 11%, TV 6%. Only five women (3%) reported STI symptoms.
4YBY, Iwelunmor et al 2022	Nigeria	Youth aged 14-24 across Nigeria	Public health/primary care	Both services integrated into a third context	HIV self testing with information to link youth to youth-friendly health clinics for STIs testing and treatment	syphilis, NG, CT	NA	NA	NA
ANRS 12381 PRINCESSE, Becquet et al 2021	Cote d’Ivoire	Adult women engaging in sex work in 19 HIV hotspots in Uganda	Mobile clinic/venue-based	Both services integrated into a third context	Syndromic STI screening, dysplasia testing (+ treatment when necessary), STI treatment	Syphilis, NG, CT	NAAT for GC,CT; Syphilis rapid test	Syndromic screening and etiological testing and treatment baseline then quarterly up to 24 months	NA
Anza Mapema, Mehta et al 2021	Kenya,	HIV negative MSM in Kisumu, Kenya	Study clinic	STI services within PrEP program	GC/CT testing and treatment; syndromic management.	Urethral and/or rectal NG, CT	NAAT for NG, CT	Urethral, and/or rectal CT NG at baseline, 6- and 12- months in urine, and rectal swabs; genital and rectal examination for signs of STIs every 3 months	The prevalence of urethral CT and/or NG infection at baseline was 10.3% (95% CI 6.0–16.2%), decreasing to 7.7% at 6 months (95% CI 4.0–13.1%), and increasing to 10.9% (95% CI 6.4–17.1%) at 12 months. Incidence of urethral CT/NG was 18.5 cases/100 person-years; incidence of rectal CT/NG was 19.9 cases/100 PY
CohMSM-PrEP, De Baetselier et al 2019	Mali, Cote d’Ivoire, Burkina Faso, Togo,	Adult MSM at risk for HIV	Public health/primary care	STI services within PrEP program	STI testing	NG, CT in rectal, urine, and pharynx, TV in urine, and Mycoplasma genitalium (MG) in rectal, urine, and pharynx	NG, CT and TV: NAAT; MG: S-DiagMGTV multiplex assay	At baseline	Chlamydia prevalence was 17.9% (12.3% anorectal, 5.7% urethral) anorectal. Gonorrhea prevalence was 15.8% (10.7% anorectal, 5.7% pharyngeal) MG infection was 26.0% for the Lomé and 27.6% for the Ouagadougou site. Only 1 participant was positive for TV.
CohMSM-PrEP, Laurent et al 2021	Mali, Cote d’Ivoire, Burkina Faso, Togo,	Adult MSM at risk for HIV	Public health/primary care	STI services within PrEP program	Screening and treatment, testing for GC, CT, and syphilis; provision of condoms, testing.	Urethra, rectum, and pharynx NG/CT, syphilis	NG, CT: NAAT; syphilis: treponemal and non-treponemal serology assays	GCCT was tested at months 0, 6, and 12 using urine, anorectal, and pharyngeal samples; syphilis every 3 months in the first year of the follow-up, and once a year thereafter	7% had STI symptoms at baseline; prevalence of gonorrhoea was 12·6% (73 of 578) at month 0, 11·2% (45 of 402) at month 6, and 14·0% (47 of 336) at month 12. The respective figures were 19·3% (65 of 336), 15·9% (37 of 232), and 20·0% (40 of 200) for chlamydia, and 0·2% (1 of 597), 0·2% (1 of 414), and 1·0% (3 of 340) for syphilis.
Community PrEP Study, Peters et al 2021	South Africa,	AGYW at community-based PrEP program in the Eastern Cape, South Africa	Community-based	STI services within PrEP program	STI testing	NG, CT, TV	NAAT for NG, CT	6-, 12- and 24-months visits; didn’t specific specimen type;	STI test positivity increased from 23 to 30% for Chlamydia trachomatis, 7% to 14% for Neisseria gonorrhoeae, and 8 to 12% for Trichomonas vaginalis
DREAMS, Chabata et al 2021	Zimbabwe,	Young women who sell sex aged 18-24 years	Community-based	Both services integrated into a third context	syndromic management of STIs	NA	NA	NA	Accessed STI treatment services in the past 12 months at the enrollment: 67/74 (90.5) in the intervention area and 75/93 (80.6) in the non-intervention area. STI symptoms in the last 12 months: 188/963 (19.5) in DREAMS cities, 206/896 (23.0) in non-DREAMS cities
ECHO, Beesham et al 2020	Eswatini, Kenya, South Africa and Zambia,Multiple Africa	HIV- women aged 16–35 years seeking long-acting reversible contraception, randomly assigned to copper intra- uterine device, intramuscular depot medroxyprogesterone acetate, or levonorgestrel implant	Sexual and reproductive health	Both services integrated into a third context	STI testing and treatment, offering condoms	Not specified	Not specified	Not specified	Prevelanc in women who initiated PrEP vs those who did not: CT = (127 (20.4%) vs. 475 (15.8%)); NG 121/3004 (4.0%) vs. 33/622 (5.3%)
ECHO, Beesham et al 2021	South Africa,	HIV negative women, aged 16 to 35 years	Sexual and reproductive health	Both services integrated into a third context	STI testing, treatment and partner notification of STIs; condom provision	Not specified	NAAT	endocervical swabs at enrollment and final visit	25% had chlamydia and 2.3% had gonorrhea detected at enrolment
Healthy Families PrEP Study, Chitneni et al 2020	Uganda	HIV negative women, 18 to 40 years old, in serodicordant relationship or with partner of unknown serostatus, with plans to conceive	Sexual and reproductive health	Both services integrated into a third context	STIs screening and treatment; partner notifications; patient-delivered partner medications;	Syphilis, NG, CT, and TV	syphilis: a rapid immunochromatographic test (ICT) confirmed by RPR; NG, CT and TV: NAAT	syphilis: blood sample; GCCT and TV: vaginal swabs. STIs were tested at enrollment.	24% had at least 1 STI including 13% with chlamydia, 2% with gonorrhea, 6% with TV, 6% with syphilis, and 3% with STI coinfection. All STI cases received treatment. 96% received partner notification cards, and 84% received patient delivered partner medications
HPTN 082, Celum et al 2021	South Africa and Zimbabwe	HIV negative women aged 16-25 at risk for HIV	Study clinic	STI services within PrEP program	STIs testing and treatment for those who were tested positive.	Syphilis, NG, CT, and TV	syphilis: RPR followed by a treponemal-specific confirmatory assay; NG, CT: NAAT; TV: rapid test (OSOMTrichomonas Test)	syphilis: blood sample; GCCT and TV: vaginal swabs. STIs were tested at enrollment, 6 months and 12 months.	Rates of STIs were 29% for chlamydia, 8% for NG, 7% TV and <1% for syphilis
HPTN 082, Delany-Moretlwe et al 2019	South Africa, Zimbabwe	HIV negative women 16 to 25 years, at risk for HIV	Study clinic	STI services within PrEP program	STIs testing and treatment for those who were tested positive.	Syphilis, NG, CT, and TV	syphilis: RPR followed by a treponemal-specific confirmatory assay; GCCT: NAAT; TV: rapid test (OSOMTrichomonas Test)	syphilis: blood sample; GCCT and TV: vaginal swabs. STIs were tested at enrollment, 6 months and 12 months.	At baseline 9% of women had CT, 8% GC, 7% TV and 2% reactive syphilis serology. STI incidence was 29.5 per 100 PY for CT, 12.2 per 100 PY for GC, and 6.9 per 100 PY for TV
IMARA-SA, Donenberg et al 2021	South Africa	15–19-year-old Black South African AGYWs and their female caregivers	Study clinic	Both services integrated into a third context	Education on factors that affect HIV/STIs risk; STIs testing and treatment	NG, CT and TV	NA	baseline, 6-, and 12-month	NA
MP3, Sullivan et al 2020	South Africa	MSM and transgender women in Cape Town and Port Elizabeth	Public health/primary care	Both services integrated into a third context	STIs testing and treatment	Syphilis, NG, CT	syphilis by RPR testing and titres and T pallidum particle agglutination; urethral and rectal CT and NG by PCR testing	blood and urine, and rectal swabs collected at baseline, 6- and 12-month visits	-
MP3, Jones et al 2020	South Africa	MSM and transgender women in Cape Town and Port Elizabeth	Public health/primary care	Both services integrated into a third context	STIs testing and treatment	Syphilis, NG, CT	Syphilis RPR testing and titres and T pallidum particle agglutination; urethral and rectal CT and NG by PCR testing	blood and urine, and rectal swabs collected at baseline, 6- and 12-month visits	Baseline: universal acceptance of urethral (292/292; 100%) screening, near-universal acceptance of syphilis (289/292; 99%) screening, and 189 (64.7%) accepted rectal STI screening. Among those screened, 29 (10%) had urethral CT infection and 8 (3%) had urethral NG; 47 (25%) had rectal CT and 30 (16%) had rectal NG, 50 (18%) had prevalent syphilis. Incident urethral CT was 12.8/100 PY and the rate of incident urethral NG was 7.1/ 100 PY. Incident rectal CT: 33.4/100 PY; Incident rectal NG 26.8/100 PY. The rate of incident syphilis infection was 8.2/100 PY. 91%, 95% and 97% of rectal, urethral, and syphilis infections clinically asymptomatic
MyPrEP, Seidman et al 2021	South Africa	HIV negative women between 18 and 25 years at a public health clinic	Public health/primary care	Both services integrated into a third context	STI testing	NG, CT	Not specified	at screening	37% of participants has NG or CT
Partners PrEP, Celum et al 2014	Kenya and Uganda	HIV- men and women in serodiscordant partnerships	Study clinic	STI services within PrEP program	STIs testing	HSV-2	EIA	At baseline	
Partners Scale-Up, Irungu et al 2021	Kenya	Patients at 25 public health clinics in Kenya at risk for HIV	Public health/primary care	STI services within PrEP program	medical assessment and syndromic STI evaluation	Not specified	Not specified	Not specified	STI assessment at 87% of follow up visits
POWER, Travill et al 2021	South Africa	young women, ages 16–25, in South Africa	Mobile clinic/venue-based	Both services integrated into a third context	STI screening and treatment	NG, CT	NAAT	At baseline	34% had any curable STI, 27% CT, 3% NG and 3% both; 65% with an STI were successfully contacted and treated
POWER, Stewart et al 2019; Rousseau et al 2021	Kenya	AGYW aged 16–25	Sexual and reproductive health	Both services integrated into a third context	STI screening and treatment	NG, CT	NAAT	Urine sample was tested at baseline and 6 monthly	48% (259) tested positive for an STI (NG and/or CT. 17% prevalence of CT and 8% of NG at enrollment. At 6 month follow up, 40.0 per 100 PY cases of chlamydia and 12.3 per 100 PY cases of gonorrhea
PREP-PP, Joseph Davey et al 2021	South Africa	HIV- pregnant women >15 years at first ANC visit	Maternal and child health	Both services integrated into a third context	STI testing	NG, CT, TV	NAAT	At baseline	35% were diagnosed with an STI
PrIYA, Kinuthia et al 2020	Kenya	HIV- women >15 years at MCH clinics in Kenya	Maternal and child health	Both services integrated into a third context	syphilis screening and syndromic STI management	syphilis and other STIs	RPR testing for syphilis; assays for other STI not specified	syphilis at baseline; syndromic STI management	1.5% of participants had previously been diagnosed with an STI
Safer Conception Intervention for Partners, Heffron et al 2019	Kenya	HIV serodiscordant couples with fertility desires in Kenya	Sexual and reproductive health	Both services integrated into a third context	STI testing and treatment	NG, CT, TV	Hologic Aptima Gen-probe	diagnostic test was provided monthly	8 people (out of 74 couples) were infected with chlamydia, 2 with gonorrhea and 3 with trichomonas
Sakh’umndeni, Schwartz et al 2017	South Africa	HIV-affected individuals of reproductive age in relationships in which one or both partners are HIV + and who want to have a child within 6 months	Sexual and reproductive health	Both services integrated into a third context	syphilis screening and syndromic STI management; treatment for those positive	syphilis and other STIs	RPR testing for syphilis; assays for other STIs not specified	syphilis was tested at baseline; monthly follow-up visits for syndromic STI management	Prevalence of symptomatic STIs at enrolment was 4% among women and 3% among men
Sakh’umndeni, Iyer et al 2019 Sakh’umndeni, Schwartz et al 2019	South africa,	Couples with at least one HIV-positive partner desiring pregnancy	Sexual and reproductive health	Both services integrated into a third context	syphilis screening and syndromic STI management; treatment for those positive	syphilis and other STIs	RPR testing for syphilis; assays for other STIs not specified	syphilis was tested at baseline; monthly follow-up visits for syndromic STI management	Reported STI history, baseline and incident STI detection *via* syndromic diagnosis through follow up for women was 3% (*n* = 9/334), 3% (*n* = 11/334) and 12% (*n* = 21/182) among women retained through six months of follow-up. Syndromically treated STI at enrollment: 11/334 among women, 5/192 among men; Syphilis diagnosis: 3/334 among women, 1/192 among men. No STI outcomes at follow up visits reported
Senegal PrEP Demonstration Project Roberts et al 2020; Sarr et al 2020	Senegal,	Adult HIV-negative women sex workers in Dakar, Senegal	Public health/primary care	Both services integrated into a third context	free condoms, STI testing and treatment, and counseling	syphilis, NG, CT	Abbott Real Time NG/CT assays, and rapid plasma reagin with Treponema pallidum agglutination assay confirmation for syphilis	STIs testing baseline, months 1, 3, 6, 9, and 12. but due to logistical constraints limited STIs testing was conducted	40% received gonorrhea and chlamydia testing at least once during the study period, 7.5% tested positive for gonorrhea and 7.5% tested positive for chlamydia. 15.4% of 221 tested women had a positive T. pallidum. Prevalence of syphilis was 1.5%, CT 6.1%, and NG.6%
TAPS Demonstration Project, Eakle et al 2017	South Africa	FSWs at two public health clinics	Public health/primary care	Both services integrated into a third context	free condoms, STI testing and treatment, and counseling	syphilis, NG, CT,	NA	Didn’t specify the testing frequency; blood samples for syphilis	STIs were assessed by syndromic management. At baseline, 17/224 participants were diagnosed with an STI. 3, 3, 2 and 5 additional STIs were diagnosed at 3 months, 6 months, 9 months and 12 months of follow up, respectively
TDF2 study Gust et al 2016	Botswana,	Heterosexual men and women at risk for HIV in Botswana	Study clinic	STI services within PrEP program	free condoms, STI testing and treatment, and partner notification	syphilis, HSV2, NG, CT, TV, BV, Candida spp.	syphilis: Determine® Syphilis TP (Abbott); HSV2: HerpeSelect® IgG ELISA (Focus); Wampole® Impact RPR; HSV2: ELISA; hepB: ELISA; GCCT: COBAS® AMPLICOR PCR Analyzer; TV: TV culture Saline Wet Prep; BV: Premixed Gram stain, BVBlue rapid; Candida spp: KOH wet prep	syphilis: Whole blood from fingerstick at Enrollment visit and annually; HSV2: Whole blood from phlebotomy at Enrollment visit; hepB: Whole blood from phlebotomy at Screening visit; GN, CT, TV: Cervical/vaginal swab (women) or Urine (men) at baseline and every 6 months; Candida spp: Vaginal or penile glans swab.	
VOICE, Marrazzo et al 2015	South Africa, Uganda, and Zimbabwe,Multiple Africa	Women 18-45 years, not pregnant, at risk for HIV	Study clinic	STI services within PrEP program	STI testing, condoms	syphilis, hepB, NG, CT, TV, BV, Candida spp.	NG, CT: strand-displacement amplification assay; TV: OSOM Trichomonas Rapid Test; syphilis: rapid plasma reagin screening test followed by a confirmatory microhemagglutinin assay for Treponema pallidum or a T. pallidum hemagglutination assay for reactive samples; HSV2: Herpe Select 2 enzyme immunoassay; BV: Nugent score	enrollment, annually, and when indicated. Vaginal fluid was collected for BV other didn’t specify.	Baseline revalence: CT 12%; NG 3%, TV 6%, syphilis 1%, HSV-2 46%, BV 40%. 10% chlamydia, 6% trichomonas, 3% gonorrhea, 1% syphilis of reported 10% chlamydia, 6% trichomonas, 3% gonorrhea, 1% syphilis). In the TDF/FTC group: 14% chlamydia, 6% trichomonas, 5% gonorrhea, 1% syphilis among those reporting SAE
Peer-Delivered HIVST, STI Self-Sampling and PrEP for Transgender Women in Uganda 2020	Uganda	Transgender women 14 years or older at risk for HIV	Community-based	Both services integrated into a third context	peer delivered STI Self-Sampling	NA	NA	NA	
Kitenge et al 2021	South Africa	HIV negative women 18 to 35 years old at community HIV testing sites in KwaZulu Natal	Community-based	Both services integrated into a third context	STI screening and treatment	NA	NA	STI testing was conducted at 3-, 6- and 12-month follow-up visits	
Heffron et al 2021	South Africa	18 and 25 years old, willing to use contraception (condoms included), HIV and Hepatitis B, uninfected,and not pregnant or breastfeeding.	Sexual and reproductive health	Both services integrated into a third context	STI screening and treatment	syphilis, NG, CT	RPR tests for syphilis, and Aptima GenProbe or GeneXpert for GCCT	Blood sample for syphilis and urine sample for GCCT; one time testing	Chlamydia in 18.2%, gonorrhea in 2%, syphilis in 0%
Behanzin et al 2021	Kenya	Women 15-30 years old, receiving care for pregnancy loss at 3 Kenyan facilities	Sexual and reproductive health	Both services integrated into a third context	STI testing	HBV HCV	immuno-chromatographic test was used for detecting HBV surface antigen (HBsAg, active infection) and HCV antibodies in whole blood. Enzyme immunoassays were used for detecting HBV core (anti- HBc) and surface (anti-HBs) antibodies	blood sample was collected once	Prevalence of active and lifetime HBV were 8.8% and 37.7%, respectively. 0.98% of participants were positive for HCV
Wahome et al 2020	Benin	MSM from the community-based PrEP demonstration study in Cotonou, Benin	Community-based	STI services within PrEP program	syndromic STI management	NG, CT, syphilis	Positive RPR titre confirmed by Treponema pallidum haemagglutination assay (TPHA) for syphilis; NG, CT was diagnosed by detection of Gram-nega-tive, intracellular diplococci	baseline and quarterly follow up; blood sample for syphilis and urethral or rectal secretions for NG, CT	Only 1 participant (0.6%) had STI at baseline (testing for syphilis and gonorrhea done)
Oluoch et al 2021	Kenya	AGYW in Kenya	Public health/primary care	STI services within PrEP program	STI testing	NG, CT, TV	NA	quarterly with STI testing	12% of AGYW tested positive for an STI.
Cassidy et al 2021	Kenya	NA	Public health/primary care	STI services within PrEP program	syndromic STI management and treatment	NA	NA	month 1 and 2 visits, and thereafter returned every two or three months	10/72 STI positive among enrolled, 19/164 among PrEP initiated; In the first six months of follow-up, 16 women (27.6%) had a syndromic STI, decreasing to 8 (13.8%) in the final six months
Masyuko et al 2018	Kenya	Adult MSM at risk for HIV in Mtwapa town	Study clinic	STI services within PrEP program	NA	NA	NA	NA	The key indicators for routine tracking iin the PrEP monitoring and evaluation program include number diagnosed with STI
Medina-Marino et al 2022	South Africa	AGYW from study clinic	Study clinic	STI into PrEP program	Baseline testing for CT, NG, TV and syphilis	CT, NG, TV, syphilis	CT, NG, TV NAAT, rapid syphilis testing	baseline; vaginal swab	At enrolment, 227 (37.6%) participants had a positive STI test result, of which 134 (59.0%) were asymptomatic. Chlamydia trachomatis (CT, *n* = 182; 30.6%) accounted for the highest-burden STI, followed by Neisseria gonorrhoeae (NG, *n* = 59; 10.0%), Trichomonas vaginalis (TV, *n* = 42; 7.1%) and syphilis (*n* = 2, 0.3%)
Velloza et al 2022	South Africa	AGYW from youth focused HIV clinic in Johannesburg	Youth-focused HIV clinic	STI into PrEP program	Etiologic testing and treatment of curable STIs (GCCT, syphilis,TV) at enrolment, 6 months and study exit	NG, CT, TV, syphilis	NA	enrolment, 6 months and study exit; specimen type not specified	NA
Beesham et al 2022	Durban, South Africa	AGYW in sexual and reproductive health clinics	Sexual and reproductive health	PrEP/STI into third context	STI testing at baseline	NG, CT	NAAT	At baseline; swabs	At PrEP initaiton, 33 and 3 women were diagnosied with Chlamydia trachomatis and Neisseria gonorrhoea respectively.
Stewart et al 2022	Kenya	HIV negative, non-pregnant young women (18-20 yo) using PrEP research clinic in Kisumu	Research clinic	STI into PrEP program	treatment arm: doxycycline as STI prevention strategy; treatment and control arm: STI screening (Chlamydia trachomatis, Neisseria gonorrhoeae, and Treponema pallidum) and treatment and risk-reduction counseling without dPEP	NG, CT, and Treponema pallidum; for future testing: staphylococcus aureus, bv, MG, and vaginal microbiome	NG, CT & TV: NAAT; syphilis: RPR or fourfold increase in non-treponemal titers;	At baseline, 3 months blood sample; endocervical swabs	NA
Davey et al 2022	South Africa	Pregnant and breastfeeding women Antenatal care	Antenatal care	PrEP/STI into third context	Baseline CT, NG, TV etiologic testing and treatment	GC, CT, TV	Point of care PCR testing	Baseline; vaginal swab	30% STI diagnosed at baseline; 14% STI diagnosed and treated same day
Ndenkeh et al 2022	Cameroon	FSW and MSM in drop-in centers of the participating community-based organization partners	Community-based organization	STI into PrEP program	monthly STI screening	STI (not specified); syphilis	TPHA and VDRL tests for syphilis	monthly; specimen type not specified	Not being reported
Skovdal et al 2022	Zimbabwe	AGYW in clinics in Eastern Zimbabwe	community in ann urban suburb and a rural village	PrEP/STI into third context	General SRH services	NA	NA	NA not specified	NA
Rutstein et al 2022	Malawi	STI clients that are eligible for PrEP use; STI clinic staff	STI clinic	PrEP into STI program	STI testing and treatment	NG, CT, syphilis	GCCT: NAAT; syphilis: rapid plasma regain, Treponema pallidum particle agglutination;	baseline, 3 months after; urine and blood samples	NA
Chidumwa et al 2022	South Africa	Young men and women aged 16-29 years from public health/primary care; community-based	Community-based public health/primary care clinics	PrEP/STI into third context	Etiologic testing; syndromic management	NG, CT, TV	samples are sent to AHRI laboratories to be processed	baseline; urine and blood samples	NA
Moran et al 2022	South Africa	Pregnant and breastfeeding women Antenatal care	Antenatal care	PrEP/STI into third context	Baseline CT, NG, TV etiologic testing and treatment	NG, CT, TV	Point of care PCR testing	baseline vaginal swab	STI prevalence of CT, NG and/or TV was 35% at first ANC visit
Mantell et al 2022	Kenya	Male clients of female sex workers, HIV negative aged at least 18-year-old in Kisumu County	Clinical reserach site	STI into PrEP program	STI testing at baseline and refered for the treatment	NG, CT, trich	specific method did get specified	baseline; urine sample blood and urine samples	NA
Mayanja et al 2022	Uganda	AGYW from GWHP clinic in Uganda	GWHP clinic serving AGYW in Uganda	STI into PrEP program	STI testing	CT, NG, syphilis	GCCT NAAT, rapid syphilis, HBV serology	3 months; Endo-cervical swabs	26.9% STI at enrolment
Inghels et al 2022	Eswatini	Clients visiting 6 public sector healthcare facilities that provide free ART and PrEP servcies	public sector healthcare facilities	PrEP into STI program	existing STI care	Not specified	Not specified	Not specified	Lower PrEP uptake was found for individuals seeking antenatal care (30.5%, 64/210), STI (30.9%, 29/94) and family planning visits (31.0%, 84/271); majority of people seeking STI services preferred PrEP delivery at outpatient services (66.7%)
Celum et al 2022	Kenya and South Africa	AGYW seeking family planning and SRH service and primary care and eligible for PrEP use	family planning clinics, mobile clinics, and public primary healthcare	STI into PrEP program	STI testing and treatment at baseline and 6 months	NG, CT	NAAT	baseline and 6 months; specimen type not specified	29% had chlamydia and10% gonorrhoea at baseline; The incidence of C. trachomatis and N. gonorrhoeae was 42.9/100 person-years (95% CI 37.2, 49.2)—35.4/100 person-years for C. trachomatis (95% CI 30.3, 41.2) and13.0/100 person-years for N. gonorrhoeae (95% CI10,0,16.7)
Mansoor et al 2022	South Africa	AGYW from study clinic	Study clinic	STI into PrEP program	STIs (NG, CT and TV) and BV testing were performed at enrolment and study exit. STI treatment and contact tracing	CT, NG, TV	NAAT	At baseline and study exit; genital swab	STI in 22.4%, BV in 56.4%
Medina-Marino et al 2023	South Africa	AGYW from study clinic	Study clinic	STI into PrEP program	Baseline testing for CT, NG, TV and syphilis	CT, NG, TV, syphilis	CT, NG, TV NAAT, rapid syphilis testing	At baseline; vaginal swab	At enrolment, 227 (37.6%) participants had a positive STI test result, of which 134 (59.0%) were asymptomatic. Chlamydia trachomatis (CT, *n* = 182; 30.6%) accounted for the highest-burden STI, followed by Neisseria gonorrhoeae (NG, *n* = 59; 10.0%), Trichomonas vaginalis (TV, *n* = 42; 7.1%) and syphilis (*n* = 2, 0.3%)

Prevalence of STIs varied between studies. Baseline prevalence of genitourinary CT, NG, TV ranged from 5.6–30.8%, 0.0–11.2%, and 0.4–8.0% respectively. Baseline prevalence of syphilis ranged from 0.0–18.0%. Baseline prevalence of symptomatic STI by syndromic screening ranged from 0.0–11.6%. STIs detected by routine laboratory testing were reported to be frequently asymptomatic. In one study of MSM receiving PrEP, 91%, 95% and 97% of rectal, urethral and syphilis infections were clinically asymptomatic (11). In one study of AGYW receiving PrEP, baseline STI prevalence was 32%, though only 3% reported symptoms (12), while another study of AGYW presenting for HIV testing services reported 38% STI prevalence at baseline, of which 59% were asymptomatic (13). Etiological testing frequency ranged from monthly to baseline testing only.

A few distinct models of integration of STI services with PrEP emerged from our review. These included routine integration of etiologic STI testing within PrEP RCTs, routine integration of syndromic STI management within public health clinics offering PrEP, etiological screening for STIs within PrEP programs targeting at-risk groups, and community outreach and mobile-based approaches. Of these, novel approaches to integration of PrEP and STI services included use of mobile clinics and other youth-focused approaches to increase convenience and acceptability (14, 15), and provision of STI testing results as a tool for improving retention in PrEP (16). However, in most studies STI diagnosis and treatment were offered as part of a standard package of PrEP services without a focus on improving either service through integration.

Few studies described quantitative or qualitative outcomes relating to implementation of integrated PrEP and STI services. Qualitative interviews from one study evaluating mobile provision of sexual and reproductive health services including STI and PrEP services found that AGYW appreciated the practicality of service integration (14). In contrast, healthcare providers in another study cited stigma as a reason not to integrate PrEP with other sexual health services (17). There was some evidence of synergy between PrEP and STI services. One report demonstrated improved uptake of PrEP services when results of STI testing was made available to AGYW (18). Several studies demonstrated an association between positive STI diagnosis and PrEP acceptance (19, 20). Implementation science research relating to STI and PrEP service integration was very limited. One study described logistical constraints limiting availability of laboratory STI testing, but these were not explored in detail (21). One research protocol described a planned study comparing standard of care syndromic management with community-based STI services including etiologic testing, but no results are yet available (22).

## Discussion

This scoping review describes existing literature around integration of STI and PrEP services in SSA. Prior literature describing provision of both these services covers a range of implementation settings, from public health clinics, sexual and reproductive health settings, mobile clinics and in the community. Most studies targeted particular at-risk groups, such as AGYW, FSW or MSM. Studies largely occurred within an existing service setting or within a PrEP program, but in nearly all studies, PrEP was the primary focus.

Despite a growing interest and rollout of PrEP in SSA, and an ongoing epidemic of curable STIs, we found limited literature covering the integration of PrEP and STI services from an implementation science perspective. Few studies addressed barriers and facilitators of integration of PrEP and STI programs. Prior qualitative work has demonstrated that challenges facing PrEP implementers in incorporating STI services include financial barriers to laboratory STI testing, logistical barriers to STI treatment in mobile settings, time constraints, lack of equipment and lack of training and capacity building around STI services(7). However, our review found a lack of evidence base addressing these barriers. In addition, while a few studies indicated potential synergies between STI and PrEP services in terms of service uptake and client satisfaction, this was not explored in detail. Most studies reported results of STI testing when offered as part of PrEP services, but did not explore the effect of bundling these services on outcomes such as acceptability or adoption.

Types of STI services offered varied widely between studies and settings, reflecting the different funding and implementation environments between RCTs, demonstration projects, and studies of real-world implementation. Some studies reported diagnostic STI screening only at baseline, while others conducted routine screening at regular intervals, and others only performed syndromic managements of STIs. The intervals of routine screening, when performed, was variable, as were the types of STIs screened. Current national PrEP guidelines in countries such as Kenya, Uganda and South Africa do not offer detailed guidance on how STI screening should be conducted (3–5), including standard guidance regarding which STIs to screen, site and frequency of screening in different populations.

Of note, laboratory diagnostic testing of STIs was mainly limited to well funded clinical studies and demonstration projects, with limited evidence from real-world public health settings. Where reported, STI prevalence and incidence by laboratory testing were high among most PrEP cohorts (11, 14, 17, 19, 23–29), with the exception of two studies of safer conception cohorts, which reported low rates of laboratory-diagnosed STIs (30, 31). In many settings, STIs diagnosed by laboratory screening were asymptomatic (12, 26, 33), reaffirming the pressing need to incorporate etiological diagnostics in STI programs offered within PrEP services.

Our scoping review has limitations. Due to the nature of the scoping review, reviewers did not assess study quality but instead gathered existing knowledge on STI services offered with PrEP programs. The review may not have captured all studies related to this topic, including sources of grey literature beyond NGO and IGO reports searched in our review. Furthermore, studies published in languages other than English were not included. Our review included abstract and study protocols in order to capture emerging literature given the relative novelty of study on the topic of PrEP and STI integration; the data collected from these sources may be less reliable than traditional peer-reviewed articles. Given the rapid emergence of literature around PrEP, our review may have failed to capture most recently published or presented literature on this topic.

Despite growing acknowledgement of the limitations of syndromic management (2), which is currently the standard practice in SSA regarding STI services within PrEP programs, few studies have addressed real-world integration of expanded STI services within PrEP programs in SSA. Future studies should examine how inclusion of additional STI services affects acceptability, adoption, and client satisfaction with both PrEP and STI services. In addition, studies should investigate cost, feasibility, and best practices regarding implementing etiologic STI diagnosis and treatment into PrEP programs in real world settings. Finally, more work in needed to understand practice setting and population-specific needs regarding integration of STI and PrEP services, including what services best suit key populations such as AGYW, MSM, FSW, and heterosexual couples, as well as how to best offer PrEP services within existing STI service contexts.

## Conclusions

In this scoping review, we found a range of STI services integrated with PrEP programs in SSA. STIs are common among populations using PrEP in SSA, highlighting the need for integration of these services. There was some evidence that integration of STI and PrEP services improves uptake of and satisfaction with both PrEP and STI services but more rigorous studies are needed to describe synergies, barriers and best practices for integration of PrEP and STI programs in SSA.
