# Supplementary material for: Integration of sexually transmitted infection and HIV pre-exposure prophylaxis services in sub-Saharan Africa: a scoping review
Source: Front Reprod Health. 2023 Jun 29;5:944372. doi: 10.3389/frph.2023.944372 (PMC10338918; doi:10.3389/frph.2023.944372)
Supplement: Supplementary file 1 [file Table1.docx]

**Table 1: Summary of included studies**

| **Study features** | **N (%)** |
| --- | --- |
| **Study Design** |  |
| RCT (including cluster RCT) | 12 (19.7%) |
| Cohort | 34 (55.7%) |
| Cross-sectional | 4 (6.6%) |
| Case report | 1 (1.6%) |
| Qualitative | 1 (1.6%) |
| Protocol only | 9 (14.8%) |
| **Year of publication** |  |
| 2022 | 18 (29.5%) |
| 2021 | 18 (29.5%) |
| 2020 | 10 (16.3%) |
| 2019 | 8 (13.1%) |
| 2018 | 2 (3.3%) |
| 2017 | 2 (3.3%) |
| 2016 | 1 (1.6%) |
| 2015 | 1 (1.6%) |
| 2014 | 1 (1.6%) |
| **Region** |  |
| Southern | 30 (49.2%) |
| Eastern | 15 (24.6%) |
| Western | 12 (19.7%) |
| Multiple | 4 (6.6%) |
| **Population studied** |  |
| AGYW | 20 (32.8%) |
| Women | 8 (13.1%) |
| PBFW | 3 (4.9%) |
| Couples | 6 (9.8%) |
| FSW | 7 (11.5%) |
| MSM | 7 (11.5%) |
| Trangender women | 1 (1.6%) |
| Multiple | 4 (6.6%) |
| Youth | 2 (3.3%) |
| Other/General | 3 (4.9%) |
| **Context** |  |
| Public health/primary care | 16 (26.2%) |
| Sexual and reproductive health | 14 (23.0%) |
| Study clinic | 14 (23.0%) |
| Community based | 7 (11.5%) |
| Maternal and child health | 5 (8.2%) |
| Mobile clinic/venue-based | 2 (3.3%) |
| Multiple | 3 (4.9%) |
| **Integration direction** |  |
| Both services integrated into a third context | 34 (55.7%) |
| STI services within PrEP program | 24 (39.3%) |
| PrEP services within STI program | 3 (4.9%) |
| **STI services described (n = 44)^1^** |  |
| Laboratory testing at baseline | 33 (75.0%) |
| Laboratory testing at follow up | 20 (45.5%) |
| Syphilis testing only | 2 (4.6%) |
| Syndromic management | 13 (29.6%) |
| Referral for treatment | 3 (6.8%) |
| STI treatment | 30 (68.2%) |
| Partner treatment | 2 (4.6%) |
| **STIs and related conditions tested (n=39)^2^** |  |
| Chlamydia | 30 (76.9%) |
| Gonorrhea | 31 (79.5%) |
| Trichomonas | 17 (43.6%) |
| Syphilis | 21 (53.9%) |
| Bacterial vaginosis | 4 (10.3%) |
| Candida | 1 (2.6%) |
| Herpes simplex | 3 (7.7%) |
| Mycoplasma genitalum | 2 (5.1%) |
| Hepatitis B | 12 (30.8%) |
| **Testing modality (n=39)^2^** |  |
| Syphilis serology | 13 (29.6%) |
| Syphilis rapid test | 8 (18.2%) |
| GC NAAT | 19 (43.2%) |
| Gonorrhea gram stain | 1 (2.3%) |
| TV NAAT | 6 (13.6%) |
| TV rapid test | 4 (9.1%) |
| Wet mount miscroscopy | 2 (4.6%) |
| Mycoplasma genitalum multiplex assay | 1 (2.3%) |
| HBV serology | 7 (15.9%) |
| HbsAg rapid test | 5 (11.4%) |
| Not specified | 11 (25.0%) |
| **Routine testing frequency (n=39)^2^** |  |
| Baseline only | 18 (40.9%) |
| Annual | 1 (2.3%) |
| 6 months | 12 (27.3%) |
| 3 months | 6 (13.6%) |
| Monthly | 2 (4.6%) |
| Other | 2 (4.6%) |

1. Out of 44 unique study reports (excluding duplicates and studies not reporting specific STI results)
2. Out of 39 unique studies conducting laboratory/etiologic testing for STIs (5 studies reporting sydromic screening only were excluded)
3. For studies with multiple screening intervals or different screening intervals for different STIs, the shortest interval of screening was used. For example, routine testing at 6, 12 and 24 months was counted as a 6-month interval of screening.

GC = N. gonorrheae and C. trachomatis; TV = T. vaginalis; HBV = hepatitis B; HbsAg = hepatitis B surface antigen; NAAT = nucleic acid amplification test

AGYW = adolescent girls and young women; FSW = female sex workers; MSM = men who have sex with men; PBFW = pregnant and breastfeeding women
